# Farther Remarks on the Spontaneous Evolution of Children, Presenting with the Arm at the Time of Birth

**Published:** 1784

**Authors:** 


V. *Farther Remarks on the spontaneous evolution of children, presenting with the arm at the time of birth. Communicated in a letter to* Samuel Foart Simmons, M.D. F.R.S. *by* Thomas Denman, M.D. *Licentiate in Midwifery of the Royal College of Physicians, Physician-man-midwife to the Middlesex Hospital, and Teacher of Midwifery in London.*

YOU did me the favour of inserting in the first number of the London Medical Journal for 1784*, an account of the spontaneous evolution of children presenting with the arm at the time of birth. I trust it will appear in that paper, that, as far as it depended upon

* See page 64.

my my own experience, I have been ſufficiently careful to aſcertain the fact, and to prevent any inferences which could be prejudicial to patients under ſuch circumſtances. I now take the liberty of requeſting you to add the following extracts from letters ſent to me on that ſubject. The letters are written by gentlemen of high character in the profeſſion, for their integrity, ability and experience, and may be conſidered as affording a full confirmation of the truth of the obſervation which I had the pleaſure of communicating to you.

---

Extract of a letter from Dr. Cogan, late Phyſician to the Charity for delivering Poor Women at their own Habitations.

" In the preſentation of children with the " ſuperior extremities, it has always been con" cluded, that every pain, which the mother " had, increaſed the difficulty; that the lower " the child was protuded into the *vagina* the " more immoveably it would be fixed; and " that there was no poſſibility of ſaving the " life of the woman, but by turning the child,

" and delivering by the feet. You were the " first who taught us to expect, that, even " under these circumstances, Nature, by the " exertion of her own powers, is able to ex- " tricate herself, by first moulding and ad- " justing the child to the *pelvis*, and then ex- " pelling it. I am fully convinced of the " truth of your doctrine, and have the plea- " sure of sending you two cases selected from " many others, which, in my opinion, place it " beyond any reasonable doubt.

" In January 1773, a midwife belonging to " the charity sent for me to a woman who had " twins. The first child was born, but the se- " cond presented with the arm. Another en- " gagement prevented my immediate attend- " ance; but I went within the hour, and when " I arrived at the place, I found, to my great " surprise, that the second child was already " born, but dead. The midwife informed me " that the pains had been exceedingly strong, " and had finished the labour without any as- " sistance; and though the arm had presented, " that the child came footling into the world. " Examining the child, I observed that the " right arm was very much swollen, that the " blood had settled upon the ribs, and that the

" position

" position in which it could be placed with the " greateſt eaſe, ſeemed moſt correſpondent " with ſuch preſentation.

" In June 1776, I was deſired by a young " gentleman to viſit, with him, a perſon who " lived near Aldgate, to whom he had been " called by a midwife. He informed me, that " the arm of the child was protruded very low, " and that he had made many attempts to turn " the child, but without ſucceſs, on account of " its great ſize and the violence of the pains.

" I was convinced, by an examination, that " his repreſentation of the poſition of the child " was truly ſtated. Whilſt we withdrew to " deliberate upon the method in which we " ſhould proceed, and to give the patient a " little reſpite after the long and painful at- " tempts which had already been made, the " labour advanced ſo kindly, that when I ſat " down I was able to hook my finger in the " groin of the child, when by very gentle aſ- " ſiſtance, the child was preſently expelled by " the feet."

---

Extract

### Extract of a letter from Dr. Patrick Hair, at Lisbon.

" I have received your aphorisms on preter-

" natural presentations, and very much regret,

" that I had not them some years ago. They

" would have given me authority and courage

" to have pursued a method, by which the life

" of a very deserving woman might have been

" saved. Of the spontaneous evolution of

" children presenting with the arm, you have

" now had many instances. My experience

" has supplied me with three, which were un-

" doubted proofs of the truth of what you have

" advanced upon that subject, and of these

" I will send you a particular account, if you

" think it will answer any good purpose."

---

### Extract of a letter from Mr. Hey, F. R. S. surgeon at Leeds.

" I must now give you the history of a deli-

" very, in which a lusty child was expelled by

" the natural efforts of the mother, after the

" arm and shoulder had been pushed down into

" the *vagina*, and the *uterus* was so closely and

" ſtrongly contracted, that it was impoſſible to
" introduce the hand in order to bring down the
" feet.

" The membranes had been broken ſix days,

" during which period the woman had ſlight

" labour pains. The midwife, whoſe at-

" tendance had not been conſtant, firſt per-

" ceived the right arm to be fallen into the

" *vagina* in the beginning of the ſixth day;

" for when the membranes broke, no part of

" the child could be felt. A young practitioner

" had been called in, as ſoon as the poſition of

" the child was known. He gave the patient

" a mixture, with ſome *tinctura thebaica*, fre-

" quently in the courſe of the day, and in the

" afternoon made an attempt to turn the child,

" but the labour pains being ſtrong and fre-

" quent, he found the delivery impracticable.

" I firſt ſaw the woman at four in the after-

" noon, and in a ſhort time attempted to carry

" my hand into the *uterus* in the moſt ſlow and

" cautious manner, but I was obliged to deſiſt,

" finding the operation impoſſible with any

" force which I dared to exert, and that I was

" unable to endure the very ſtrong compreſſion

" upon my hand by the contracting *uterus*.

" As

" As confiderable efforts to deliver had been
" made before my arrival, I was afraid, left
" thefe renewed attempts fhould caufe an in-
" flammation of the *uterus*, and therefore de-
" fired the gentleman, who was yet attending
" the patient, to take away fome blood, and to
" give forty drops of *tinctura thebaica*. I then
" left the woman for an hour, during which
" time fhe was kept in a ftate of perfect tran-
" quillity, and drank nothing but balm tea or
" cold milk and water.

" At my return, I made another attempt to
" deliver, but the ftricture at the *collum uteri*
" was abfolutely unyielding, and I was again
" obliged to defift. Twenty drops of *tinctura*
" *thebaica* were now given to her, and fhe was
" fuffered to reft for another hour, in which
" time the pains went off and fhe flept.

" Though every circumftance now feemed
" very favourable for the delivery, yet I found,
" upon making a third attempt, that the ftric-
" ture before mentioned, would not give way
" to any force I could fafely ufe, and I was con-
" vinced that it was impoffible to deliver by the
" feet.

" When I ceafed to make any farther at-
" tempts to deliver, the pains became exceed-

" ingly ſtrong, the ſhoulder deſcended lower

" into the *pelvis*, and I began to entertain hopes

" that the delivery would be effected by the

" natural pains, as in the caſes which you were

" ſo obliging as to communicate to me. I then

" ſuffered the woman to make her efforts with-

" out interruption. The ſhoulder was ſoon

" protruded through the external parts; the

" ſide of the child could be perceived, and the

" ſpine was incurvated; the loins then appeared,

" and the child was at length expelled by the

" feet. The placenta came away properly.

" The child was dead, but the mother reco-

" vered.

" The knowledge of what you have ſaid

" upon this ſubject was a great comfort to me,

" when I found the artificial delivery imprac-

" ticable, and I think you ought to make your

" obſervations as public as poſſible."

---

The fact of the ſpontaneous evolution being thus aſcertained, I muſt leave it to the future obſervations of other practitioners, to determine upon the particular caſes, in which that event may be reaſonably expected and properly waited waited for. Though the knowledge of the fact has already been attended with advantage to many women in very deplorable circumstances, much remains to be done to complete the doctrine, not by reasoning upon the subject, but by careful attention to practice.

I am, dear Sir,

Your obliged and most humble servant,

*20th August, 1784.* T. DENMAN.

*Old Burlington-street.*

